# The Association Between Temporomandibular Disorders and Tinnitus: Evidence and Therapeutic Perspectives from a Systematic Review

**DOI:** 10.3390/jcm14030881

**Published:** 2025-01-29

**Authors:** Gianna Dipalma, Alessio Danilo Inchingolo, Carmela Pezzolla, Roberta Sardano, Irma Trilli, Daniela Di Venere, Francesco Inchingolo, Andrea Palermo, Angelo Michele Inchingolo

**Affiliations:** 1 Department of Interdisciplinary Medicine, University of Bari “Aldo Moro”, 70124 Bari, Italy; giannadipalma@tiscali.it (G.D.); a.inchingolo1@studenti.uniba.it (A.D.I.); carmela.pezzolla@uniba.it (C.P.); roberta.sardano@uniba.it (R.S.); trilliirma@gmail.com (I.T.); daniela.divenere@uniba.it (D.D.V.); a.inchingolo3@studenti.uniba.it (A.M.I.); 2 Department of Experimental Medicine, University of Salento, 73100 Lecce, Italy; andrea.palermo@unisalento.it

**Keywords:** tinnitus, temporomandibular disorders, joint dysfunction, orofacial pain, dental malocclusion, masticatory muscles, mandibular condyle, central sensitization, comorbidities, neuromodulation

## Abstract

**Background/Objectives:** Tinnitus, often described as a ringing in the ears, affects a significant portion of the population, varying in perception and severity. **Methods:** This systematic review investigates the correlation between tinnitus and temporomandibular joint disorders (TMDs) within a PRISMA-compliant framework, ensuring methodological transparency and rigor. Using databases, such as PubMed, Scopus, and Web of Science, we analyzed studies from the past decade to evaluate clinical and observational evidence. **Results:** A significant association between TMD and tinnitus was found, with somatosensory and neuroplastic mechanisms contributing to this relationship. Key therapeutic approaches identified include manual therapy and multidisciplinary treatments, demonstrating potential clinical efficacy. **Conclusions:** However, the available evidence remains inconsistent, emphasizing the need for further research with standardized methodologies to improve understanding and refine therapeutic strategies. This review provides a foundation for future studies aiming to enhance tinnitus management by addressing underlying TMD-related mechanisms.

## 1. Introduction

Tinnitus, commonly known as ringing in the ears, is the perception of sound without an external auditory stimulus [1,2,3]. This auditory experience can vary widely, manifesting as buzzing, hissing, whistling, or a combination of sounds [4,5,6,7]. The perceived sounds can be localized in one ear, both ears, or felt centrally “in the head” [8,9,10]. The prevalence of tinnitus in the adult population ranges from 10% to 15%, with a significant increase among individuals over 60 years old, where prevalence reaches up to 18% [11,12].

### 1.1. Types of Tinnitus and Pathophysiological Mechanisms

Tinnitus can be classified into two main categories: objective and subjective [13,14,15]. Objective tinnitus can be heard by an external observer and is often associated with specific conditions, such as vibrations in the auditory system or vascular abnormalities [16,17,18]. However, most cases of tinnitus are subjective, heard only by the patient [19,20,21]. Subjective tinnitus is frequently attributed to neuroplastic changes in the central auditory system in response to peripheral auditory system damage [22,23,24]. Recent studies suggest that damage to the peripheral auditory system, such as acoustic trauma, can alter spontaneous neural activity, leading to increased excitability of central auditory neurons [25,26,27]. These changes are considered part of a compensatory neuroplasticity process that may contribute to the persistent perception of tinnitus [28,29,30].

### 1.2. Influence of the Somatosensory System on Tinnitus

A particularly relevant aspect of subjective tinnitus is its modulability through the somatosensory system [31,32,33,34,35]. In about two-thirds of patients with subjective tinnitus, the perception of sound can be modified by muscle contractions or movements of the neck, head, or jaw [36,37,38,39]. This phenomenon, known as somatosensory tinnitus, suggests that the somatosensory system can influence tinnitus perception and implies that alterations in muscular or articular structures can impact its intensity and tone [40,41,42]. Somatosensory influences are believed to act through cross-modal interactions between the auditory and somatosensory pathways within the brainstem, particularly involving the dorsal cochlear nucleus, which receives input from both systems. This anatomical and functional connection provides a theoretical framework explaining how cervical and temporomandibular dysfunctions might modulate tinnitus perception [43,44].

### 1.3. Association Between Tinnitus and Temporomandibular Disorders (TMDs)

Temporomandibular disorders (TMDs) encompass a heterogeneous group of conditions affecting the temporomandibular joints and masticatory muscles. These disorders are frequently linked to tinnitus, indicating a potential association between the two conditions.

**Figure 1:**
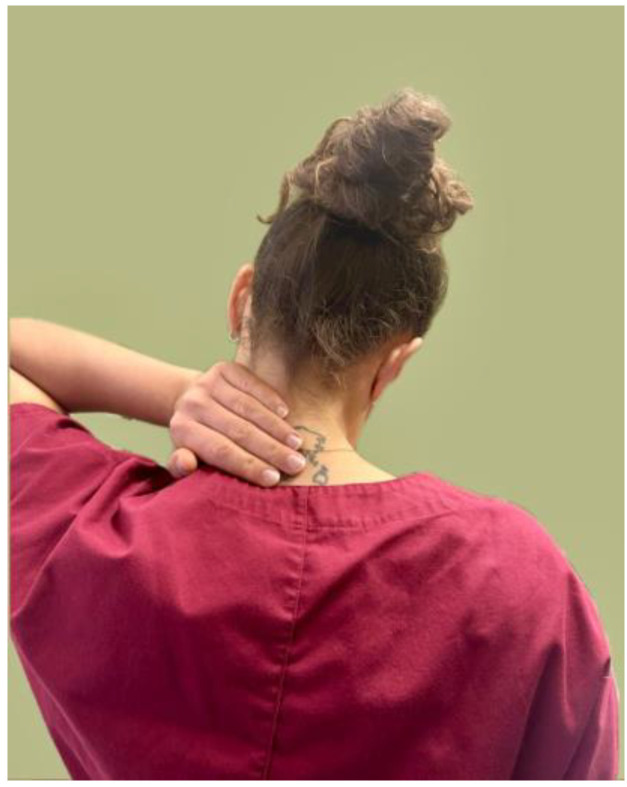
Tinnitus can be influenced by myofascial pain, particularly in the masticatory and cervical muscles.

TMD can affect individuals across all age groups, with the highest prevalence observed among those aged 20 to 40 years. However, TMD has also been documented in children and adolescents, and it has been noted that the primary non-dental cause of orofacial discomfort in this population is TMD-related pain [45,46]. Symptoms of TMD, such as pain, restricted mandibular movement, and altered masticatory function, may influence tinnitus perception through somatosensory mechanisms (Figure 1) [47,48,49]. Clinical and observational studies have demonstrated a frequent co-occurrence of tinnitus and TMD, although the strength and nature of this relationship remain uncertain [50,51,52,53,54]. Some evidence suggests that treatment targeting TMD could alleviate tinnitus symptoms, although results are inconsistent and dependent on study design and therapeutic approaches [55,56,57,58,59]. Further exploration of this link through robust research is necessary to clarify causality and therapeutic potential [60,61].

### 1.4. Cervical Disorders and Their Impact on Tinnitus

Cervical spine disorders (CSDs), encompassing conditions like neck pain and cervical pain syndrome, have also been linked to tinnitus [62]. Symptoms of CSD, including pain and stiffness in the cervical region, may affect tinnitus perception through somatosensory pathways [63,64,65,66]. Research suggests that neck movements and improper postures can modulate tinnitus perception, highlighting a potential interplay between cervical pain and tinnitus symptoms. However, the strength and consistency of the evidence are limited by variability in diagnostic criteria and study methodologies. Further investigation is essential to determine precise mechanisms and effective interventions [67,68,69].

### 1.5. Need for a Systematic Review

Despite extensive research investigating the associations between tinnitus, TMD, and cervical spine disorders (CSDs), a comprehensive systematic review analyzing these relationships is lacking [70,71]. Many existing studies suffer from limitations, including inadequate control groups and inconsistent assessment methodologies for evaluating the strength of associations.

Addressing these gaps requires a systematic review to consolidate current knowledge and assess the bidirectional relationship between subjective tinnitus and symptoms of CSD or TMD [72,73,74].

### 1.6. Objectives of the Review

This systematic review aims to critically evaluate the literature on the relationship between subjective tinnitus and both temporomandibular disorders (TMDs) and cervical spine disorders (CSDs) [75,76]. The primary objective is to determine the strength of evidence supporting bidirectional associations between these conditions, drawing insights from clinical and observational studies. Additionally, it seeks to identify methodological gaps and propose avenues for future research, with a focus on therapeutic implications and the potential for targeted interventions to improve tinnitus management.

## 2. Materials and Methods

### 2.1. Protocol and Registration

The PRISMA (Preferred Reporting Items for Systematic Reviews and Meta-Analyses) protocols were followed when conducting this review, and the protocol was registered at PROSPERO under the CDR ID 641352.

### 2.2. Research Process

We used PubMed, Scopus, and Web of Science as online databases, in which we searched for publications that matched the topic of the review. The search strategy was developed using specific keywords and Boolean operators to target studies relevant to the correlation between TMD and tinnitus in adults. The exact search terms included “tinnitus”, “TMJ”, and the Boolean operator “AND”. An example search string was “tinnitus AND TMJ”. The inclusion of only English-language articles was a practical limitation due to resource constraints and the prevalence of key publications in English, but this choice may introduce publication bias. Articles published between October 2014 and October 2024 were considered, which were chosen to capture the most recent decade of research, reflecting contemporary advancements and clinical practices (Table 1).

### 2.3. Inclusion Criteria

The inclusion criteria were (1) studies on humans, (2) open access, (3) English language (English abstracts and foreign language texts were found), and (4) articles related to adult populations.

### 2.4. Exclusion Criteria

The following exclusion criteria were applied: (1) case reports, (2) animal studies, (3) in vitro studies, (4) reviews, (5) off-topic articles, and (6) pediatric trials. To ensure methodological rigor and transparency, the inclusion and exclusion criteria for this systematic review were developed following the PICO (Population, Intervention, Comparison, Outcome) framework. This structured approach helps define the scope of the review and ensures that the selected studies align with the research objectives. To support the use of the PICO framework, the methodology has been outlined in previous studies [72] (Table 2).

### 2.5. Data Processing

Articles were excluded if they did not align with the topic after reviewing the title and abstract. Full texts of the remaining articles were thoroughly examined to determine relevance according to the inclusion criteria. Any disagreements among authors regarding article selection were resolved through discussion and consensus.

### 2.6. Quality Assessment

The quality of included studies was evaluated using the Cochrane Risk of Bias Tool for randomized controlled trials and the ROBINS-I tool for non-randomized studies, ensuring a rigorous assessment of study reliability and potential biases.

## 3. Results

### 3.1. Selection and Characteristics of the Study

A total of 976 publications were identified through online databases: PubMed (*n* = 675), Scopus (*n* = 251), and Web of Science (*n* = 50). No additional studies were identified through manual searches. After removing 211 duplicate records, 765 studies remained and were screened by title and abstract. Among these, 642 studies did not meet the inclusion criteria, leaving 26 records for further consideration. A total of 123 reports were requested for retrieval, with none missing.

**Figure 2:**
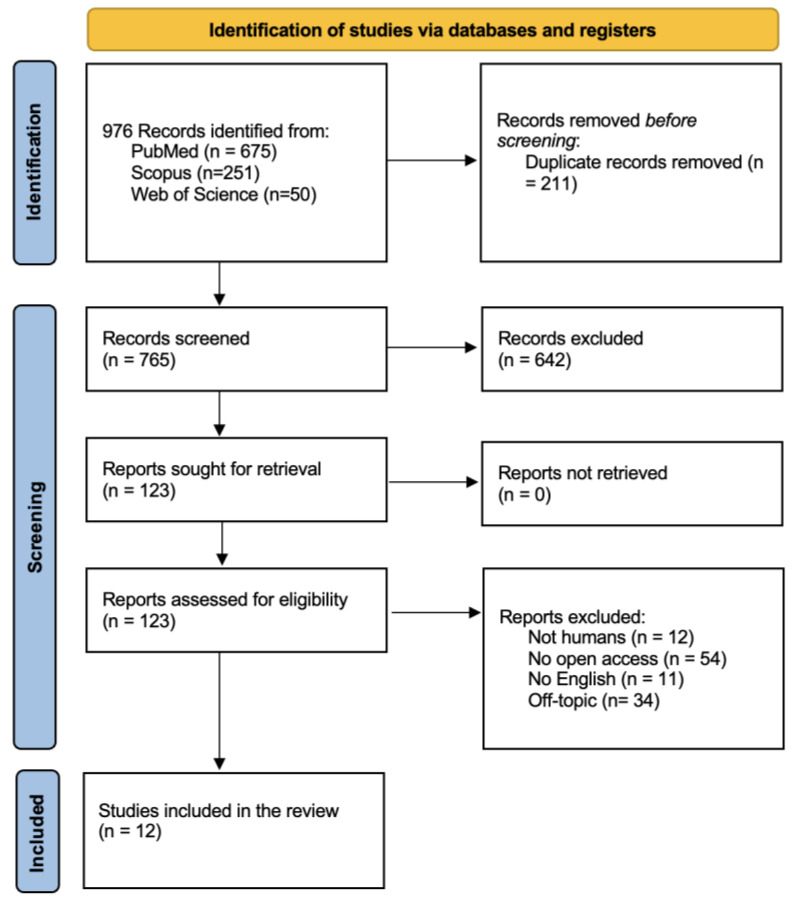
PRISMA flow chart.

Following a detailed eligibility assessment of the 123 reports, 111 were excluded for not meeting the criteria, leaving 12 studies for qualitative analysis. The selection process and the summary of included records are illustrated in Figure 2, while the characteristics of the selected studies are presented in Table 3, which includes a column evaluating study quality and risk of bias using the Cochrane Risk of Bias Tool for randomized trials and the ROBINS-I tool for observational studies.

### 3.2. Risk of Bias Assessment in Included Studies

**Figure 3:**
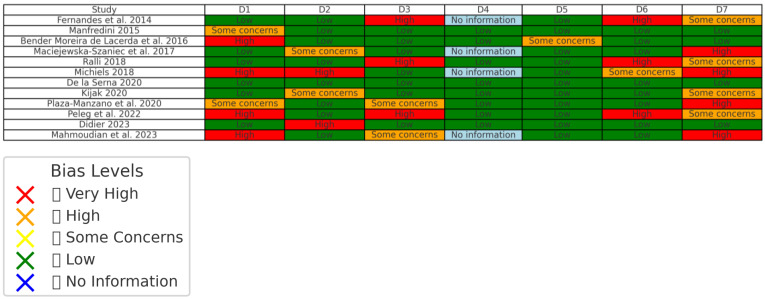
Risk of bias ([56,78,79,80,81,82,83,84,85,86,87]).

The risk of bias assessment table evaluates the included studies across seven key domains. Most studies showed “some concerns” or a “low risk” of bias in various domains, indicating moderate overall quality. Common areas with “higher risk” were related to “confounding factors (D1)” and “selection bias (D3)”, reflecting challenges in controlling variables and participant selection processes. In contrast, “measurement of outcomes (D6)” was generally well-handled, with most studies rated as having a “low risk” of bias, demonstrating reliable methods for assessing key variables like tinnitus and TMD outcomes. Some studies lacked sufficient information in specific domains, leading to “uncertainties (D7)” about reporting bias. Overall, while the table highlights methodological strengths in outcome measurement and analysis, it also underscores areas needing improvement, particularly in study design and participant selection (Figure 3).

## 4. Discussion

### 4.1. Prevalence and Correlation of TMD and Tinnitus Symptoms

The relationship between temporomandibular disorders (TMDs) and tinnitus has been extensively documented in the literature. Maciejewska-Szaniec et al. [79]. conducted in 2017 a clinical study on 246 TMD patients (aged 40.08 ± 11.12 years, 147 females, 99 males) that explored the link between TMD and otologic symptoms (OS). The study found that 36.18% (n = 89) had OS. These patients were divided into two subgroups: G1.1 (46.07%, n = 41) with audiological abnormalities and G1.2 (53.93%, n = 48) without abnormalities. Ear fullness and otalgia were more common in G1.2, while hearing impairment was more frequent in G1.1. Tinnitus was observed in 14.63% of the total group, and hypersensitivity to sounds in 10.57%. The findings suggest the need for subjective hearing loss assessment in TMD patients with OS, as these symptoms often coexist with TMD and may indicate more advanced dysfunctions. Additionally, clicking and popping noises in the jaw were more common in patients without hearing impairment. The study highlights the importance of considering OS in the diagnosis and treatment of TMD to ensure comprehensive patient care [79].

Moreover, Peleg et al., 2022 [85] explored the coexistence of temporomandibular disorder (TMD) and bruxism among 51 tinnitus patients. Participants completed the Tinnitus Handicap Inventory (THI), Beck’s Depression Inventory (BDI), the State Anxiety Inventory (SAI), and the Diagnostic Criteria for TMD (DC/TMD). Clinical evaluations included oral cavity, facial muscle, and temporomandibular joint (TMJ) assessments. The sample comprised 27 males and 24 females, aged 23–77. Bruxism was diagnosed in 34 patients (66.7%) and TMD in 14 patients (27.5%). Those with tinnitus, TMD, and bruxism had the highest anxiety scores. A significant correlation was found between tinnitus severity and depression. No gender differences were observed in TMD and bruxism prevalence.

Most patients with TMD also had bruxism (92.8%). Additionally, tinnitus severity was negatively correlated with age. The study supports the theory of somatic tinnitus and suggests that TMD and bruxism exacerbate tinnitus severity and psychological distress. The findings emphasize the importance of multidisciplinary evaluation for tinnitus patients to address coexisting TMD and bruxism, thereby improving overall patient care and avoiding misdiagnosis [85].

Fernandes et al.’s 2014 [56] cross-sectional study sought to investigate the connections among temporomandibular disorders (TMDs), tinnitus, and sleep bruxism (SB) in 261 women (mean age 37.0 years) seeking treatment for orofacial pain. The Research Diagnostic Criteria for Temporomandibular Disorders were used in the study to classify TMD, and clinical criteria were used to diagnose SB. The findings showed that tinnitus and painful TMD were significantly correlated (OR = 7.3; 95% CI = 3.50–15.39; *p* < 0.001).

In contrast, SB had a weaker association with tinnitus (OR = 1.9; 95% CI = 1.16–3.26; *p* < 0.0163). Notably, individuals with painful TMD without SB showed a strong correlation with tinnitus (OR = 6.7; 95% CI = 2.64–17.22; *p* < 0.0001), while those with both conditions experienced more severe tinnitus (OR = 7.0; 95% CI = 3.00–15.89; *p* < 0.0001). The findings suggest that while SB is linked to TMD, it does not directly correlate with tinnitus. The study highlights the necessity of additional research to understand these associations and highlights the importance of addressing TMD management to alleviate related symptoms [56].

Manfredini et al. [77] investigated the correlation between tinnitus and TMD symptoms in 250 patients, analyzing the presence of masseter muscle pain, internal derangement, and TMJ osteoarthritis. About 30.4% of TMD patients reported tinnitus, with a tendency for higher prevalence among those with masseter muscle pain and TMJ osteoarthritis. Age-stratified analyses indicated significant associations between tinnitus and specific TMD symptoms in different age groups. The results suggest possible symptom-specific links and highlight the need for further studies with more objective assessments [77].

Didier et al. [86] examined the prevalence and relationship between somatosensory tinnitus and TMD in 97 patients with somatosensory tinnitus and 50 with TMD symptoms. The study found a strong association between TMD and tinnitus, with TMD diagnosed in 97.8% of patients. The high rates of popping, clicking, and pain in patients highlight the need for further research with larger samples and specific screening tools to explore the relationship between tinnitus and TMD [86].

In 226 patients with chronic tinnitus, Ralli et al. investigated the connection between TMD and tinnitus. Patients with and without a history of somatic modulation of tinnitus and TMD dysfunction were separated. The study found that a history of TMD dysfunction and tinnitus modulation indicates a possible TMD in tinnitus patients. However, no direct correlation between TMDs and tinnitus origin was found. The study concludes that somatic dysfunction and tinnitus modulation may help identify patients with TMDs.

### 4.2. Treatment Efficacy and Patient Outcomes

The therapeutic management of TMD has shown promising results in alleviating tinnitus symptoms. The study by Mahmoudian et al., 2023 [87] examined six male patients with chronic non-pulsatile tinnitus and a mean age of 45.34 ± 9.57 years who had not received any treatment for three months prior. Patients had no history of neurological conditions or head injuries, confirmed by audiologists and otorhinolaryngologists. Evaluations included audiological assessments and psychoacoustic measurements of tinnitus. Tinnitus pitch and loudness matching, minimum masking level (MML), and residual inhibition (RI) were assessed.

Following TMD therapy, significant improvements in tinnitus parameters were observed. The Steigerwald/Maher TMD Disability Index (SMTDI) scores significantly decreased from 34.50 ± 1.3 to 24.37 ± 8.6 (*p* = 0.0001). Visual analogue scale (VAS) scores for tinnitus intensity, annoyance, and awareness significantly improved (*p* < 0.05). Audiometric thresholds showed no significant differences pre- and post-treatment (*p* > 0.05). After therapy, clicking and crepitus improved for 7.1 ± 0.9 months, and bruxism ceased in three patients. The study concludes that TMD therapy may alleviate tinnitus in individuals with somatosensory tinnitus and concurrent TMD symptoms, suggesting the benefits of a multidisciplinary treatment approach [87].

A secondary analysis of a clinical trial was conducted by Plaza-Manzano et al., 2020 [84] to evaluate the predictive influence of various clinical, psychological, and psychophysical factors on the results of treatment for people with temporomandibular disorders (TMDs) who have tinnitus. The study involved 61 participants who were randomly assigned to either an exercise and education program or the same program combined with cervico-mandibular manual therapy. The outcomes, including tinnitus severity and related handicap, were assessed at baseline, 3 months, and 6 months post-intervention.

Higher baseline tinnitus severity scores were found to be significant predictors of improved outcomes at three and six months following the intervention, accounting for 13% to 41% of the variation in outcomes. Higher initial tinnitus-related handicap scores were linked to better outcomes in the manual therapy group, explaining 45% of the variation. On the other hand, worse clinical outcomes in both treatment groups were linked to lower baseline pressure pain thresholds (PPTs) over the temporalis muscle, which accounted for 10.5% to 41% of the variation.

PPTs over the masseter muscle explained 5.8% of the variance in the exercise/education group, while sex and quality of life explained 6.7% of the variance in the manual therapy group. The study found that the most important predictors of clinical outcomes in patients with TMD-related tinnitus after physical therapy were baseline tinnitus severity and localized pressure pain sensitivity over the temporalis muscle, with other parameters having a less significant impact [84].

De la Serna et al. [82] conducted a multicenter, randomized study to evaluate the impact of cervico-mandibular manual therapy on somatic tinnitus related to TMD. Patients were assigned to either exercise and education (EE) or exercise, education, and manual therapy (EEMT). The study found significant improvements in pain intensity, tinnitus handicap, TMD-related disability, quality of life, depression symptoms, pain sensitivity, and mandibular range of motion in the EEMT group. The results suggest a strong link between TMD and tinnitus, recommending a comprehensive therapeutic approach. Future studies should include a control group and manual therapy to further assess these interventions’ effectiveness [82].

Arruda de Souza Alcarás et al. (2023) [78] conducted complementary research to examine how tinnitus affects patients with temporomandibular disorders (TMDs) and their quality of life. Nineteen people (mean age 53.5 years, 89.47% female) made up the sample for this quantitative, cross-sectional retrospective study. The Brazilian Tinnitus Handicap Inventory (THI), acuphenometry, pure tone audiometry, and anamnesis were all used in the data gathering process.

The findings showed that 15.79% of respondents described their tinnitus as a whistle, rain, or cricket sound, while 63.16% reported having it for less than five years. In the right ear, tinnitus was more common (42.11%) and persistent (52.63%). Bilateral mean thresholds exceeding 25 dB HL at high frequencies were found through audiometry. According to acupuncture, the average tinnitus loudness in the right ear was 21 dB SL, while in the left ear, it was 17.85 dB SL. The average pitches were 3775 Hz and 3750 Hz, respectively. A moderate influence on quality of life was indicated by the mean THI score of 37.8.

Significant correlations were found between THI scores, tinnitus duration, and frequency, suggesting that the longer the duration and the higher the frequency, the greater the impact on quality of life. These findings highlight the need for multidisciplinary evaluation and treatment approaches for TMD and associated otological symptoms [78].

### 4.3. Pathophysiological Mechanisms and Interdisciplinary Approaches

Understanding the pathophysiological mechanisms linking TMD and tinnitus is critical for developing effective treatment strategies. Kijak et al. [83] studied the association between temporomandibular disorders (TMDs) and tinnitus in 331 patients, mostly women. They found a significant correlation between the position of the petrotympanic fissure, the mandibular condyle’s dislocation, and the incidence of tinnitus. Patients with tinnitus often had posterior or intracranial condyle positions, supporting the idea that certain anatomical factors in the petrotympanic fissure may predispose TMD patients to tinnitus. Limitations of the study include the lack of a control group and specific audiometric tests. The findings highlight the need for further research to understand the mechanisms linking TMD and tinnitus for potential therapies [83].

The need for interdisciplinary approaches is underscored by Michiels et al. [81], who found that conservative treatment for TMD positively impacted somatic tinnitus symptoms, advocating for the integration of dental, psychological, and medical interventions to enhance patient care and management outcomes. They conducted a randomized clinical trial to evaluate the effectiveness of conservative treatment for TMD in patients with somatic tinnitus. Patients were divided into an immediate-start group and a delayed-start group. The study found that TMD treatment, including education on proper jaw use, relaxation techniques, and stabilization splints, significantly reduced tinnitus-related distress. The beneficial effects were maintained over time, suggesting the treatment’s long-term efficacy. The results support the connection between TMD and somatic tinnitus and the importance of a multidisciplinary approach involving dentists and physiotherapists [81].

### 4.4. Methodological Limitations, Therapeutic Approaches, and Research Recommendations

The relationship between temporomandibular disorders (TMDs) and tinnitus has been extensively studied, but several limitations are common across the literature. Many studies lack control groups, making it difficult to compare findings against a healthy population. Sample sizes in some studies are relatively small, which can limit the generalizability of the results. The cross-sectional design of several studies prevents the assessment of symptom progression over time. Additionally, the reliance on clinical criteria for diagnosing conditions like bruxism without instrumental confirmation can introduce diagnostic bias.

Moreover, the review was limited by the exclusion of non-English studies, which may have resulted in the omission of relevant international research. The reliance on a restricted timeframe for literature inclusion could further narrow the scope of findings, potentially overlooking more recent or older foundational studies.

A systematic assessment of study quality was not uniformly performed across the included literature, which may affect the robustness and reliability of the conclusions. Without a standardized approach to evaluating methodological rigor, inconsistencies in study quality may contribute to varying levels of evidence.

Another critical challenge lies in the heterogeneity of study designs and diagnostic criteria. Variability in how TMD, tinnitus, and related conditions are defined and measured complicates comparisons across studies and the synthesis of findings. The use of inconsistent diagnostic tools, differing symptom severity scales, and varied outcome measures creates additional obstacles to drawing clear, generalizable conclusions.

Finally, gender-specific samples and the absence of detailed psychosocial variable analyses further limit the applicability of findings to broader populations. Some studies also lack comprehensive audiometric evaluations, which are essential for accurately assessing hearing loss and its association with TMD.

Future research should address these limitations by including larger and more diverse sample populations, adopting longitudinal study designs, applying standardized diagnostic criteria, and implementing systematic quality assessments. This would enhance the reliability, comparability, and clinical applicability of findings regarding the complex interplay between TMD and tinnitus.

Our discussion outlines significant findings regarding the association between TMD and tinnitus, and several critical considerations require further elaboration.

Heterogeneity in Diagnostic Criteria and Methodologies: The studies reviewed vary significantly in their diagnostic criteria for both TMD and tinnitus, as well as in methodologies and participant characteristics. This variability may affect the generalizability of conclusions. Future systematic reviews or meta-analyses should adopt standardized diagnostic frameworks, such as the Diagnostic Criteria for TMD (DC/TMD), to improve the comparability and reliability of results.

Limitations of the Evidence Base: A predominance of small sample sizes, reliance on self-reported measures, and lack of control groups limit the robustness of many studies. For instance, several investigations, including those by Manfredini et al. and Didier et al., [77,86] highlight associations without control groups to verify causality. Addressing these issues in future research through well-powered randomized controlled trials would strengthen the evidence [80].

Therapeutic Approaches and Critical Appraisal: Although various interventions show promise, their quality and consistency vary. Mahmoudian et al. (2023) [87] demonstrate significant symptom relief following TMD therapy, yet their small sample size limits broader applicability. Similarly, manual therapy combined with exercise and education (De la Serna et al. [82]) presents compelling evidence for an interdisciplinary approach but requires replication with control arms [87].

Actionable Recommendations for Future Research: Priorities include conducting larger longitudinal studies to assess causal relationships, employing objective audiological measures, and integrating psychological assessments to address multifactorial influences. The use of randomized controlled trials, standardized diagnostic criteria, and comprehensive outcome measures would enhance the understanding and management of TMD-related tinnitus.

## 5. Conclusions

Research indicates a significant correlation between tinnitus and various temporomandibular disorders (TMDs), emphasizing the importance of considering otologic symptoms during TMD diagnosis and treatment. Studies consistently report a high prevalence of tinnitus and other auditory complaints among TMD patients, suggesting a potential pathophysiological link between these conditions. The co-occurrence of related conditions, such as bruxism and anxiety, further highlights the necessity of a multidisciplinary approach to patient care. This approach should incorporate dental, psychological, and medical interventions to address the complex interplay of factors influencing these disorders.

Therapeutic management:

- Conservative treatments, such as manual therapy, jaw relaxation exercises, and stabilization splints, are effective in mitigating tinnitus symptoms.
- These interventions improve the overall quality of life for patients, alleviating the distress associated with tinnitus.
- A holistic approach that considers dental, psychological, and medical aspects is essential for managing patients with TMD and tinnitus.

Future Research:

- Research with larger samples, more diverse populations, and longitudinal studies is needed.
- The standardization of diagnostic criteria and the inclusion of comprehensive audiometric assessments will improve the reliability and comparability of results.
- There is an urgent need to develop standardized clinical guidelines and evidence-based therapeutic protocols.
- Exploring the mechanisms linking TMD and tinnitus will be crucial for creating targeted therapeutic strategies.

Importance of Interdisciplinarity:

An interdisciplinary approach remains essential for effectively managing and treating patients affected by both TMD and tinnitus.

## Figures and Tables

**Table 1:** Database search indicators.

Article searching strategy	Databases: PubMed, Scopus, Web of Science
	Keywords: A “TINNITUS” and “TMJ”
	Boolean variable: AND
	Timespan: 2014–2024
	Language: English

**Table 2:** PICO framework.

PICO Element	Adults with temporomandibular disorders (TMDs) and tinnitus.
Population (P)	Investigating the correlation between temporomandibular joint (TMJ) dysfunction and tinnitus symptoms.
Intervention (I)	Adults with temporomandibular disorders (TMDs) and tinnitus.
Comparison (C)	Not explicitly defined; however, studies not focused on the TMD—tinnitus correlation were excluded.
Outcome (O)	Understanding the possible correlation between TMD and tinnitus in the adult population.

**Table 3:** Featured research in the qualitative analysis and their characteristics.

Authors and Year	Type of Study	Material and Methods	Aim of the Study	Results
Fernandes et al., 2013 [56]	Cross-sectional	261 women, mean age 37.0; RDC/TMD criteria; clinical diagnosis of SB.	To investigate the association between SB, tinnitus, and TMD.	Painful TMD and tinnitus (OR = 7.3; *p* < 0.001); SB and tinnitus (OR = 1.9; *p* < 0.0163); TMD without SB and tinnitus (OR = 6.7; *p* < 0.0001); TMD with SB and tinnitus severity (OR = 7.0; *p* < 0.0001).
Manfredini 2015 [77]	Cross-sectional study	In 250 consecutive TMD patients, the presence of self-reported tinnitus was evaluated. Clinical TMD symptoms and indicators were used in the correlation analysis to evaluate the frequency of tinnitus in TMD patients who had arthrosis, internal derangements in the TMJ, or jaw muscle pain.	To compare the prevalence of tinnitus in TMD patients affected by jaw muscle pain, TMJ internal derangements, or TMJ arthrosis.	Tinnitus prevalence in TMD patients is 30.4% and higher in patients with masseter pain (32.8%) and TMJ arthrosis (33.7%), with age-stratified correlations between pain and arthrosis.
Arruda de Souza Alcarás et al., 2023 [78]	Cross-sectional retrospective	Anamnesis, pure tone audiometry, acuphenometry, Brazilian THI.	Evaluate the impact of tinnitus on the quality of life of patients with TMD.	Tinnitus present in 63.16% for <5 years, 15.79% described as whistle/rain/cricket, right ear 42.11%, continuous in 52.63%, mean THI score 37.8 (moderate impact), significant correlation between THI, duration, and frequency.
Maciejewska-Szaniec et al., 2017 [79]	Clinical research	246 patients (147 females, 99 males, average age 40.08 ± 11.12 years) with TMD. Dental history interviews and clinical examinations.	To investigate the correlation between TMD and otologic symptoms (OS).	36.18% (n = 89) had OS. Of these, 46.07% (n = 41) had audiological abnormalities (G1.1), and 53.93% (n = 48) did not (G1.2). Ear fullness and otalgia were more frequent in G1.2, while hearing impairment was more common in G1.1. Tinnitus was present in 14.63%, and hypersensitivity to sounds was present in 10.57% of patients.
Ralli 2017 [80]	Observational study	The study involved 226 patients with normal hearing and chronic tinnitus, divided into a study group with TMJ dysfunction and positive tinnitus modulation and a control group without these criteria.	To ascertain if patients with persistent tinnitus and normal hearing may have an underlying TMJ issue if they self-report a history of TMJ dysfunction and exhibit positive modulation of tinnitus in the TMJ region.	A clinically confirmed TMJ problem was found in 131 patients (57.9%) (79.1% in the research group vs. 27.2% in the control group; *p* < 0.0001). Joint diseases affected the majority of patients (67.2%). In the study group, patients with TMJ issues were more likely to be female.
Michiels 2018 [81]	Randomized controlled trial	Patients with moderate to severe subjective tinnitus with TMD or oral parafunctions were recruited for a study. Patients were randomly assigned to early or delayed-start groups, receiving 18 sessions of conservative TMD treatment over 9 weeks.	To find a subset of tinnitus patients who benefit from TMD treatment and to examine the impact of conservative TMD treatment on tinnitus in patients who have co-occurring tinnitus and TMD or oral parafunctions in comparison to no treatment.	The study aimed to evaluate the effectiveness of TMD treatment on tinnitus complaints, but specific results are not provided in the text.
Delgado de la Serna 2020 [82]	Randomized clinical trial	Sixty-one patients with TMD tinnitus were divided into two groups: physiotherapy and manual therapy and physiotherapy alone. Primary outcomes included pain intensity, tinnitus severity, and secondary outcomes.	To investigate the effects of adding cervico-mandibular manual therapies to an exercise and educational program on clinical outcomes in individuals with tinnitus associated with TMD.	The exercise/education plus manual therapy group demonstrated better outcomes for TMD pain, tinnitus severity, THI, CF-PDI, BDI-II, PPTs, and range of motion, with no significant difference in SF-12.
Kijak 2020 [83]	Observational study	331 subjects with TMD (268 women, 63 men); imaging studies of the facial skull in the sagittal plane using volumetric imaging (FOV: 17 cm × 23 cm).	To ascertain the relationship between concomitant tinnitus in TMD patients, the position and structure of the petrotympanic fissure (PTF), and condylar displacement in the TMJ.	Ten percent of TMD patients with tinnitus with rear or intracranial-cranial condylar displacement may represent a distinct phenotype of tinnitus, suggesting TMD treatment should be included in therapeutic approaches.
Plaza-Manzano et al., 2020 [84]	Randomized clinical trial	Secondary analysis of a clinical trial with 61 subjects with TMD-related tinnitus. Participants were randomly assigned to exercise/education or exercise/education + manual therapy groups.	To assess predictors of treatment outcomes in TMD-related tinnitus patients.	Higher baseline tinnitus severity predicted better outcomes (13–41% variance). Higher baseline related handicap predicted better outcomes in the manual therapy group (45% variance). Lower PPTs over temporalis muscle predicted poorer outcomes (10.5–41% variance). Other factors: sex, quality of life, PPTs.
Peleg et al., 2022 [85]	Observational study	51 patients; THI, BDI, SAI, DC/TMD questionnaires; clinical evaluation of oral cavity, facial muscles, TMJ.	To investigate the coexistence of TMD and bruxism in tinnitus patients.	66.7% had bruxism and 27.5% had TMD; significant association between tinnitus severity and depression; highest anxiety scores in patients with tinnitus, TMD, and bruxism.
Didier 2023 [86]	Cross-sectional study	The study evaluated patients with somatosensory tinnitus and TMD at Policlinic Hospital of Milan, excluding common causes and assessing joint noise and pain symptoms.	To investigate the prevalence of TMD in patients with somatosensory tinnitus and the occurrence of somatosensory tinnitus in patients with TMD.	TMD was diagnosed in 97.8% of audiological patients, with joint noise, clenching, and pain in 14.8%, while somatosensory tinnitus was diagnosed in 24.0% patients.
Mahmoudian et al., 2023 [87]	Case series	Six male patients with chronic non-pulsatile tinnitus, evaluated using audiometry, psychoacoustic measurements, and TMD assessments, and treated for TMD.	To assess the impact of TMD therapy on tinnitus.	Significant improvement in SMTDI scores (*p* = 0.0001) and VAS scores for tinnitus intensity, annoyance, and awareness (*p* < 0.05). No significant audiometric changes (*p* > 0.05).
